# Complementary detection strategies for circulating tumor cells in breast cancer: clinical implications of combining immunofluorescence and cytopathological staining

**DOI:** 10.3389/fonc.2025.1632245

**Published:** 2025-07-23

**Authors:** Tanja Jesenko, Cvetka Grasic Kuhar, Ziva Pisljar, Simona Miceska, Veronika Kloboves-Prevodnik, Maja Cemazar

**Affiliations:** ^1^ Institute of Oncology Ljubljana, Ljubljana, Slovenia; ^2^ Faculty of Medicine, University of Ljubljana, Ljubljana, Slovenia; ^3^ Faculty of Medicine, University of Maribor, Maribor, Slovenia; ^4^ Faculty of Health Sciences, University of Primorska, Koper, Slovenia

**Keywords:** biomarker, breast cancer, circulating tumor cells, cytopathological detection, immunofluorescence-based detection

## Abstract

**Background:**

Circulating Tumor Cells (CTCs) serve as important biomarkers for disease monitoring and treatment response in patients with metastatic breast cancer. Their detection remains challenging because of their low abundance, phenotypic diversity and non-standardized mode of detection. Cytopathological Giemsa and Immunofluorescence (IF) staining can offer complementary approaches for CTC characterization. Giemsa staining enables assessment of cellular morphology, while IF allows for marker-specific identification, together providing a more comprehensive and accurate evaluation of CTCs.

**Methods:**

We developed an IF staining protocol with antibodies against Cytokeratin (CK), vimentin (VIM), and Cluster of Differentiation 45 (CD45) to distinguish epithelial, mesenchymal, hybrid and hematopoietic cells for CTC detection and characterization and compared it with cytopathologic method of detection via Giemsa staining with regard to CTC detection rates and morphological detail.

**Results:**

Study was performed on the samples of 29 heavily pretreated patients with metastatic breast cancer (median duration of metastatic disease 19.4 months). Giemsa staining enabled the detection of a higher number of CTCs compared to our IF protocol. Lower detection rate was potentially due to the loss of fragile or loosely adherent cells during methanol fixation and IF staining. Additionally, in IF-stained samples, some CTCs presented faint nuclear signals, potentially impairing their recognition. The IF staining supported the identity of CTCs detected on Giemsa-stained slides by employing a three-color antibody panel-based approach and allowed detailed phenotypic discrimination and structural analysis of CTCs, including the identification of a distinctive CK polarization pattern suggestive of a transitional state during intravasation.

**Conclusion:**

Giemsa and IF may thus be complementary rather than mutually exclusive and relying on a single detection approach could underestimate the true CTC burden. An integrative strategy combining both techniques may offer a more comprehensive view of CTC populations in metastatic breast cancer, thereby enhancing diagnostic precision.

## 1 Introduction

Breast cancer is the most common cancer in women worldwide, accounting for 2.3 million of new cases and 0.66 million of deaths in 2022 (1). Over 90% of cases are diagnosed as early stage. Despite radical locoregional treatment, one third of patients relapse with distant metastases. 54 years ago, Bernard Fisher proposed in his theory that breast cancer is a micrometastatic disease from the diagnosis and that the main route of dissemination is the blood stream (2). The research in the last two decades is focused on the detection of biomarkers in liquid biopsy indicating minimal residual disease or early detection of relapse. This could be the starting point to improve prognosis and even cure patients with metastatic breast cancer, if diagnosed early.

Metastatic breast cancer is an incurable disease, with median Overall Survival (OS) depending on the breast cancer subtype (3), being 43.3 in Hormone Receptor positive (HR+)/Human Epidermal growth factor Receptor 2 negative (HER2-), 50.1 in HER2+; and 14.8 months in triple-negative subtype, as reported in French cohort (3). Current breast cancer guidelines for monitoring patients with metastatic disease recommend physical exams, blood tests, and imaging scans (Computed Tomography (CT) or Positron Emission Tomography - PET scans) every 2-4 months, with adjustments based on individual needs (4). Cancer Antigen 15-3 (CA15-3) is a widely used serum tumor marker in breast cancer; however, it is not a part of the guidelines. Metastatic breast cancer management in the last decade has been oriented much more targeted with regards to new mutations or resistance. Liquid biopsy has revolutionized patient monitoring by enabling the noninvasive detection of Circulating Tumor Cells (CTCs) and Circulating Tumor DNA (ctDNA) from peripheral blood, with the latter approach being included in the European Society for Medical Oncology (ESMO) recommendations (5–7). This technique offers real-time insights into tumor evolution, treatment response, and minimal residual disease, overcoming the limitations of traditional tissue biopsies (5).

CTCs have emerged as particularly promising biomarkers in liquid biopsy strategies, offering notable advantages over ctDNA (8). Unlike ctDNA, which is limited to fragmented genetic material, CTCs, which are viable tumor components, allow for comprehensive analyses, encompassing morphological, proteomic, transcriptomic, and epigenetic profiling (9). This capability facilitates a deeper assessment of tumor heterogeneity and the dynamic evolution of the metastatic process, including the identification of subpopulations that may drive resistance to therapy (10). Furthermore, the potential to culture CTCs ex vivo, similar to Patient-Derived cancer Organoids (PDOs) (11), enables functional assays and drug sensitivity testing, thereby advancing personalized therapeutic approaches (12–14). Therefore, investigating CTCs offers significant advantages over analyses based solely on ctDNA by providing a richer, multidimensional view of tumor biology. However, the technical complexity involved in isolating and comprehensively characterizing these cells remains a substantial challenge and there are no standardized detection methods available for their quantification thus far.

In our previous study, we directly compared two different technologies for CTC isolation: physical and biological isolation systems (15). The primary objective was to identify CTCs on the basis of cytomorphological criteria, as assessed by an experienced cytopathologist, which would enable quick translation of CTC determination/evaluation into routine practice. Our findings indicate that the physical approach enables the isolation of CTCs with better morphological preservation than the biological approach does, facilitating their identification on the basis of established cytological features of malignancy, such as large nuclei, a high Nuclear-Cytoplasmic (N/C) ratio, scant basophilic cytoplasm, and visible chromatin structure. Additionally, the physical approach yielded a greater number of CTCs, further supporting its suitability for CTC isolation and characterization following routine cytological staining (15).

However, owing to their diversity and degeneration in the bloodstream (16), CTCs frequently exhibit signs of degeneration, including cytoplasmic blebs, villous projections, irregularities in nuclear or cytoplasmic membranes, and loss of chromatin structure (15). The altered morphology of CTCs, resulting from cellular degeneration during circulation or isolation, differs from that of tumor cells obtained via Fine-Needle Aspiration Biopsies (FNAB) and can pose a challenge for cytopathologists in performing accurate identification of CTCs (17). Furthermore, the literature addressing the morphological evaluation of CTCs via routine cytological staining methods is scarce. This requires the use of additional staining techniques to confirm their identity and distinguish them from other degenerated blood cells. Immunofluorescence (IF) staining seems to be the most suitable approach, allowing simultaneous detection of multiple cell markers. The most widely used markers include epithelial markers (Cytokeratin, CK; Epithelial Cell Adhesion Molecule, EpCAM), mesenchymal markers (Vimentin, VIM; N-cadherin), and negative selection blood lineage markers (Cluster of Differentiation 45, CD45) (18–21). These marker combinations enable not only the distinction of CTCs from other blood cell types but also the characterization of their phenotype, which can be Epithelial (E), Mesenchymal (M), or a hybrid Epithelial/Mesenchymal (E/M) state and determine important markers for therapy selection (20). Notably, the hybrid phenotype is frequently observed in CTC clusters and is strongly associated with enhanced metastatic potential (22).

Therefore, the aim of this study was to develop an IF staining protocol for the detection and characterization of CTCs in peripheral blood samples from patients with metastatic breast cancer and to compare its performance with that of routine Giemsa staining in terms of the CTC detection rate and morphological evaluation. For this purpose, an IF staining protocol was established staining CK, VIM and CD45 followed by nuclear counterstain in positive control samples of MCF7 breast cancer cell line and FNAB samples from a lymph nodes, which was later validated in samples of CTCs isolated from the peripheral blood of patients with metastatic breast cancer.

## 2 Materials and methods

### 2.1 Study design

This study was conducted in two phases: the development of an IF staining protocol and its subsequent application to clinical samples. The IF protocol was initially optimized using the MCF7 breast cancer cell line to establish reliable CK staining and a FNAB sample from a lymph node to optimize staining for VIM and CD45. The reproducibility of the protocol was confirmed in three independent experiments. Following successful optimization, the protocol was applied to stain CTCs obtained from patients with metastatic breast cancer. In parallel, a corresponding patient sample was stained via the routine Giemsa method. CTCs were analyzed on slides stained via both methods and compared in terms of CTC enumeration and morphological assessment. The Giemsa staining protocol used in this study follows the standard procedure routinely applied in clinical diagnostic practice at the Department of Cytopathology, Institute of Oncology Ljubljana. The clinical study protocol, IF staining procedure, and IF image analysis workflow were independently established and optimized. The criteria for CTC identification on Giemsa-stained slides were adopted from our previous study (15).

### 2.2 Patients and control FNAB samples

Twenty-nine patients with metastatic breast cancer were enrolled in this noninterventional, prospective study, irrespective of disease duration, treatment line, or therapy cycle. The study was conducted at the Institute of Oncology Ljubljana, Slovenia, and received ethical approval from both the Institutional Research Ethics Committee (ref. no. ERIDNVPO 0021/2020) and the National Medical Ethics Committee at the Slovenian Ministry of Health (ref. no. 0120-541/2021/3). Written informed consent was obtained from all participants. The study adhered to the principles of the Declaration of Helsinki and Good Clinical Practice guidelines. Inclusion criteria were: Metastatic breast cancer; second-line or later line of systemic therapy; radiologically and/or laboratory-confirmed disease progression; patient undergoing treatment prior to the 1st to 3rd cycle. Exclusion criteria: First-line treatment for metastatic breast cancer; second-line or later treatment beyond the 3rd chemotherapy cycle; presence of a concurrent malignancy. Blood samples were collected simultaneously with routine blood draw, via a minimally invasive procedure. Each patient provided a single 10 mL blood sample in an EDTA collection tube (BD, Franklin Lakes, NJ, USA). To maximize CTC viability, all the samples were processed within four hours after collection.

In addition, a leftover cell suspension of a sample obtained via routine diagnostic FNAB of a lymph node was used for the establishment of an IF staining protocol, and at each IF staining of samples as a positive control. Written informed consent was also obtained from this patient prior to sample aspiration.

### 2.3 Cell lines

The human epithelial breast cancer cell line MCF7 (ATCC^®^ HTB-22) was obtained from the American Type Culture Collection (ATCC, Manassas, VA, USA). Cells were cultured in Advanced Minimum Essential Medium (Advanced MEM; Gibco, Thermo Fisher Scientific, Waltham, MA, USA) supplemented with 5% fetal bovine serum (FBS; Gibco), GlutaMAX™ (100×; Gibco), and Penicillin-Streptomycin solution (Sigma-Aldrich, Merck, Darmstadt, Germany). Cell line was tested for Mycoplasma infection using MycoAlert Mycoplasma Detection Kit (Lonza, Basel, Switzerland) and was Mycoplasma free. Cultures were maintained at 37°C in a humidified incubator with 5% CO_2_ until they reached approximately 80% confluence. At confluence, the culture medium was removed, cells were washed with Phosphate-Buffered Saline (1×PBS, Gibco), and detached using 0.25% trypsin-EDTA in Hank’s Balanced Salt Solution (HBSS; Gibco). Following detachment and collection, cells were counted, and a cell suspension of 5 × 10^5^ cells in 10 mL of culture medium was prepared.

### 2.4 Isolation of cells and slide preparation

The suspensions of MCF7 cells and patient blood samples were processed via the Parsortix^®^ system (Angle, Guildford, UK) according to the manufacturer’s recommendations. Parsortix^®^ system isolates CTCs on the basis of their physical properties, such as size and deformability. The separation cassette contains a stepped structure, gradually narrowing in diameter until reaching a final gap of 6.5 µm. Therefore, all of the cells that are larger than 6.5 µm are retained and isolated. Retained cells were harvested into a 5 mL plain red-top vacutainer tube (Becton Dickinson, Franklin Lakes, NJ, USA) (approximately 200 µl) without a preharvest flush and resuspended in 3 drops (approximately 150 µl) of an in-house cell medium: 20% bovine serum albumin (SERVA, Heidelberg, Germany) and 5% EDTA (Sigma Aldrich, St. Louis, MI, USA) in PBS. The isolated cell suspension was equally distributed between two cytological slides via a cytocentrifuge (Thermo Scientific Shandon Cytospin R 4 Cytocentrifuge, Waltham, MA, USA) by centrifugation at 700 rpm for 4 min at Room Temperature (RT). The first slide was used for Giemsa staining, and the second one was used for IF staining.

### 2.5 Giemsa and IF staining

For Giemsa staining, the slides were first air-dried at RT for at least 1.5 hours, followed by methanol fixation for 30 minutes. The Giemsa working solution was prepared for use in a Leica XL automated slide stainer (Leica Microsystems, Buffalo Grove, IL, USA) by manually mixing Giemsa stock solution (Sigma-Aldrich, Merck, Darmstadt, Germany; Ref. No. 1.09204) with PBS at a 1:5 ratio to a final volume of 300 mL. After methanol fixation, the slides were transferred to the stainer, where all subsequent steps were carried out automatically. These included a 30-second rinse in distilled water, a 15-minute incubation in the Giemsa staining solution, four consecutive rinses in distilled water, and a final drying step involving baking at 30 °C. After staining, the slides were air-dried at RT for 2 hours and subsequently mounted using Tissue-Tek^®^ Glass Mounting Medium (Sakura Finetek, Torrance, CA, USA; Ref. No. 148N).

For IF staining, methanol-fixed slides were first circled using a Super HT PAP Pen (Biotium, Fremont, CA, USA) to prevent reagent leakage. The slides were washed once for 5 min in PBS and then permeabilized for 30 min at RT in permeabilization buffer, which consisted of 5% donkey serum, 0.5% Triton X-100, and 22 mg/mL glycine in PBS. The permeabilization step was omitted in the testing of the protocol without permeabilization. After permeabilization, the slides were rinsed for 5 min in blocking buffer, composed of 2% donkey serum and 22 mg/mL glycine in PBS, followed by an additional 1-hour blocking step in the same buffer at RT. The slides were incubated overnight at 4°C in a humidified, light-protected chamber with primary antibodies diluted in blocking buffer. The following primary antibody mixture was used: mouse anti-human CK pan antibody cocktail (Invitrogen, Cat# MA5-13203; dilution 1:100 or 1:200), goat anti-human anti-VIM antibody (Abcam, Cat# ab11256; dilution 1:100 or 1:200), and rabbit anti-human recombinant anti-CD45 antibody [EP322Y] (Abcam, Cat# ab40763; dilution 1:100 or 1:200). The next day, the slides were washed three times with PBS for 5 min each and incubated for 1 hour at RT in PBS containing a mixture of secondary antibodies: donkey anti-mouse Alexa Fluor^®^ 488 (Abcam, Cat# ab150105; dilution 1:500), donkey anti-goat Alexa Fluor^®^ 647 (Jackson ImmunoResearch; Cat# 705-605-147, dilution 1:500), and donkey anti-rabbit Cy3 (Jackson ImmunoResearch; Cat# 711-165-152 dilution 1:500). Following secondary antibody incubation, the slides were washed three times for 5 min each in PBS. Nuclear staining was performed with Hoechst 33342 dye (3 µg/mL in PBS, Thermo Fisher Scientific) for 10 min in the dark, followed by three additional PBS washes of 5 min each. The slides were mounted using ProLong™ Glass Antifade Reagent (Thermo Fisher Scientific), which was preequilibrated to RT for at least 1 hour. Coverslips were applied, and the samples were secured with nail polish. All steps, including incubation and washing, were performed under light-protected conditions to preserve fluorophore stability. Each staining batch included positive controls. MCF7 cells were used for CK staining, whereas routine FNAB samples were used for VIM and CD45 staining.

Imaging was performed with an LSM 800 confocal microscope (Carl Zeiss, Oberkochen, Germany). Bright field color images were taken with an Axiocam 506 color camera for detecting Giemsa-stained CTCs. The slides were analyzed by an experienced cytopathologist, and the number of CTCs was determined on the basis of morphological criteria for malignant cells, such as larger size than blood cells, larger nuclei, a high N/C ratio and scant cytoplasm. The fluorophores Hoechst 33342, Alexa Fluor 488, Cy3, and Alexa Fluor 647 were excited with lasers with excitation wavelengths of 405, 488, 561 and 640 nm, respectively. The emitted light was collected sequentially with a Gallium Arsenide Phosphide (GaAsP) detector via a variable dichroic and filters at the following wavelengths: 410–545 nm (Hoechst 33342), 565-620 (Cy3), 488–545 nm (Alexa Fluor 488), and 645–700 nm (Alexa Fluor 647). The obtained fluorescence images were visualized and analyzed with Imaris software (Bitplane, Belfast, United Kingdom). The fluorescence intensity was evaluated by dividing the sum of the fluorescence intensity analyzed via the “surface” function by the number of cells (nuclei) analyzed via the “spots” function. The CTCs in IF slides were identified with the following criteria: larger cells than blood cells with a weak to strong nuclear signal that are negative for CD45 staining and positive for CK (E phenotype), VIM (M phenotype), or both (E/M phenotype).

### 2.6 Statistical analysis

Statistical analysis and graph plotting were carried out via GraphPad Prism 10 (La Jolla, CA, USA). Statistical significance was evaluated via Student’s unpaired two-tailed t-test. The following symbols indicate statistical significance *: p < 0.05. The sample size (n) for each experiment is presented in the figure legends and represents the number of biological replicates. The agreement between the Giemsa and IF staining methods for CTC counting was assessed via Bland–Altman analysis. Distribution of data was tested for normality via Shapiro-Wilk test. For each sample, the difference between the two methods was calculated and plotted against the average of the two methods. The mean difference (bias) and the standard deviation of the differences were computed. The 95% limits of agreement were then determined by calculating the bias ± 1.96 times the standard deviation of the differences. The presence of proportional bias was evaluated by visually inspecting the trend of differences across the range of average values.

## 3 Results

### 3.1 Patient cohort characteristics

Our cohort consisted of 28 women and one man. The characteristics of the patients and cancer are presented in **Table 1**. The median age of patients at primary diagnosis of breast cancer was 49.8 years (InterQuartile Range (IQR) 45.7–61.7). A total of 20.7% of patients had primary metastatic disease (stage IV), and 48.2% had stage III A-C disease. In terms of molecular subtype, the majority (55.2%) were luminal HER2-, 27.6% were HER2+ and 17.2% were triple negative. The histology report revealed invasive carcinoma of No Special Type (NST) and grade 3 in 65.5% of patients, and the median percentage of Ki-67-positive cells was 30 (IQR 16–50). At CTC collection the median age of patients was 57.1 (IQR 50.6-46.4) years. The median duration of metastatic disease was 19.4 (6.7–39.4) months. At CTC collection, patients had a median of two metastatic locations. The most common metastatic locations were bone (57.1%), liver (44.8%) and lymph nodes (37.9%). Two patients (6.9%) had brain metastases. Two-thirds of the patients were receiving chemotherapy, and the others were receiving endocrine therapy. Almost half of the patients (48.3%) were heavily pretreated (on the 3^rd^ or later line of therapy), and 72.4% were on that treatment for at least 3 cycles.

**Table 1:** Patients’ clinical characteristics.

Variable	Number (%)
Age at primary diagnosis, years; median (IQR)	49.8 (45.7 - 61.7)
Age at CTC sampling, Years, median (IQR)	57.1 (50.6 - 68.8)
OS from diagnosis of metastatic disease, months, median (IQR)	22.9 (15.9 - 46.4)
Duration of metastatic disease, months, median (IQR)	19.4 (6.7 - 39.4)
Overall survival post CTC collection (to death) months, median (IQR)	6.1 (6.1 - 11.2)
Stage at primary diagnosis, n (%)	
IA	1 (3.4)
IIA	5 (17.2)
IIB	3 (10.3)
IIIA	5 (17.2)
IIIB	1 (3.4)
IIIC	8 (27.6)
IV	6 (20.7)
Subtype	
Luminal A/B (HER2 negative)	16 (55.2)
HER2 positive	8 (27.6)
Triple negative	5 (17.2)
Histology	
NST	19 (65.5)
ILC	2 (6.9)
Other	5 (17.2)
NST+ILC	3 (10.3)
Ki-67, %,	
median (IQR)	30 (16-50)
Grade	
1	1 (3.4)
2	6 (20.7)
3	19 (65.5)
Unknown	3 (10.3)
Number of metastatic organs	
median (IQR)	2 (1 - 2)
Metastatic location at CTC collection	
Brain	2 (6.9)
Lung	7 (24.1)
Liver	13 (44.8)
Bone	15 (51.7)
Lymph nodes	11 (37.9)
Pleura/peritoneum	8 (27.6)
Type of systemic therapy at CTC collection:	
Endocrine (± CDK4/6 inhibitors)	10 (34.5)
Chemotherapy ( ± immunotherapy or antiHER2 therapy)	19 (65.5)
Line of therapy	
1^st^ or 2^nd^ line	15 (51.7)
3^rd^ or 4^th^ line	11 (37.9)
≥5^th^ line	3 (10.3)
Cycle of therapy	
1^st^ or 2^nd^ cycle	8 (27.6)
≥ 3^rd^ cycle	21 (72.4)

IQR, interquartile range; OS, overall survival; CTC, circulating tumor cells; HER2, human epidermal growth factor receptor 2; NST, no special type; ILS, invasive lobular carcinoma; Ki-67, marker of proliferation Kiel 67.

### 3.2 Establishment of an IF protocol for CTC staining

**Figure 1:**
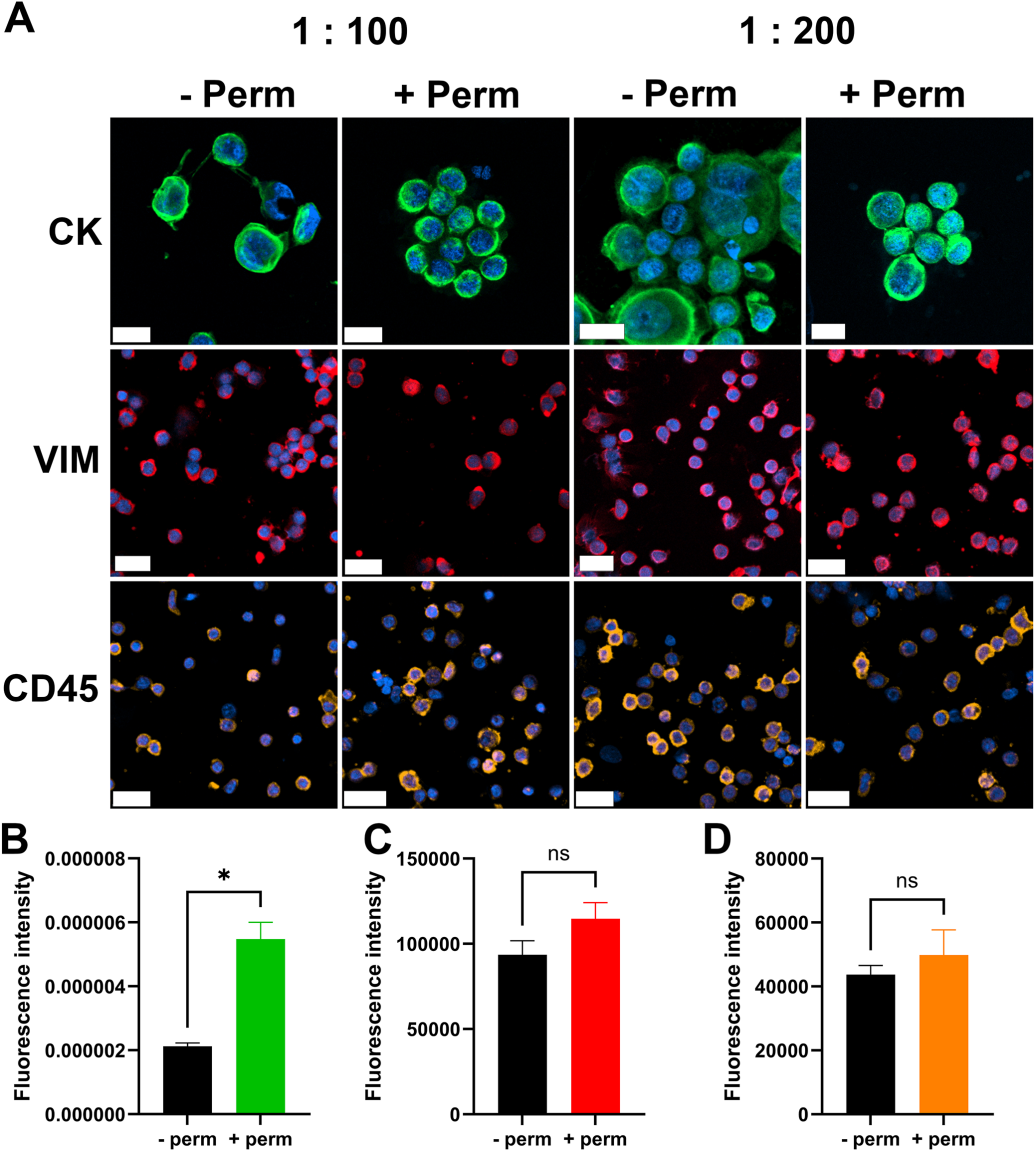
IF-staining protocol. Images of cells stained for individual cell markers **(A)**. First row MCF7 cells stained for CK, middle row lymph node biopsy sample stained for VIM, last row lymph node biopsy sample stained for CD45. Scale bar = 20 µm. CK, cytokeratin; VIM, vimentin; CD45, cluster of differentiation 45/leukocyte common antigen; -Perm, without permeabilization; + Perm, with permeabilization; 1:100 and 1:200, dilutions of primary antibody. Quantification of the fluorescence intensity of CK staining **(B)**, VIM staining **(C)** and CD45 staining **(D)**. n = 3, *p < 0.05, ns- nonsignificant.

To develop the IF staining protocol for processing slides containing CTC samples from the aforementioned patient cohort, the protocol was first optimized using cell suspensions derived from the MCF7 cell line and FNAB samples of lymph nodes. The IF protocol was a two-step staining method using primary antibodies followed by labeled secondary antibodies to increase the fluorescence signal intensity and lower the detection limit. All three antibody clones successfully stained the CK antigen in MCF7 cells and VIM and CD45 antigens in FNAB samples at dilutions of either 1:100 or 1:200 (**Figure 1A**). Therefore, 1:200 dilutions were selected for subsequent CTC staining.

Further optimization was performed to assess the necessity of a permeabilization step. We investigated whether additional permeabilization was required for effective cytoplasmic staining of CK and VIM. The permeabilization step significantly increased the fluorescence intensity of CK staining (**Figure 1B**). Cytoplasmic VIM staining and surface CD45 staining were not significantly affected by the permeabilization step (**Figures 1C, D**). On the basis of these findings, the permeabilization step was incorporated into the final protocol.

### 3.3 Populations of metastatic breast cancer CTCs in Giemsa- or IF-stained slides

**Figure 2:**
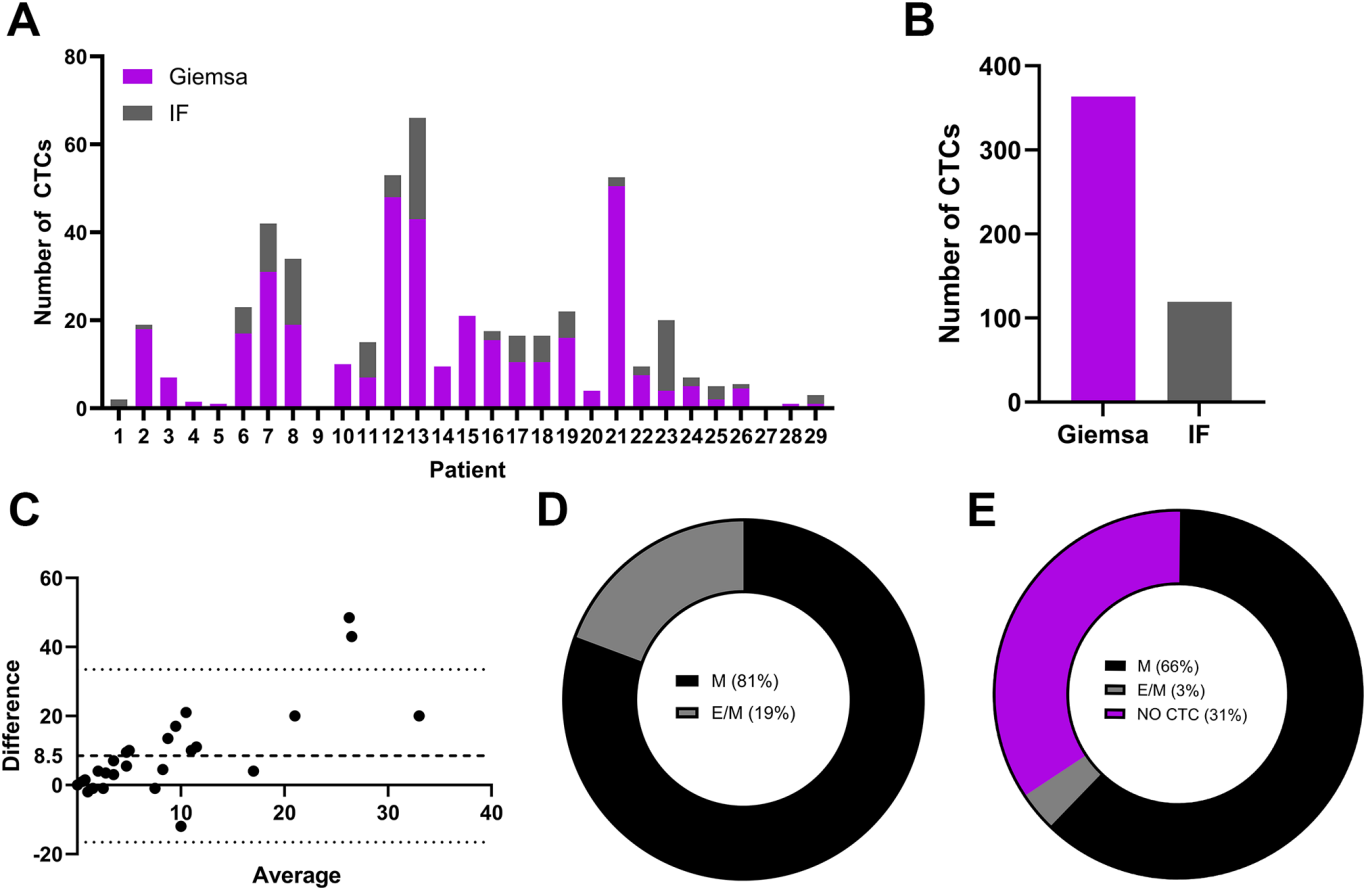
Populations CTCs from metastatic breast cancer patients on Giemsa- or IF-stained slides. The number of detected CTCs in Giemsa- or IF-stained slides from each individual patient **(A)**, the overall number of detected CTCs in a patient cohort in slides stained with Giemsa or IF protocol **(B)**, a Bland–Altman plot comparing cell counts obtained via Giemsa- and IF-stained slides **(C)**, the phenotype of CTCs as a percentage of all detected CTCs via IF staining **(D)** and the phenotype of CTCs as a percentage of patients with detected CTCs with IF staining **(E)**. IF, immunofluorescence; M, mesenchymal phenotype; E/M, hybrid epithelial/mesenchymal phenotype.

Following the establishment of IF staining protocol, the slides containing CTC samples from the patient cohort were stained and analyzed. The detection rate of CTCs via the standard cytological Giemsa staining protocol was compared with that via the IF staining protocol. The number of CTCs in the metastatic breast cancer patient cohort was determined via Giemsa- and IF-stained slides. The sample of each patient was equally distributed among the cytological slides; therefore, similar numbers were anticipated when either of the staining protocols was used. According to the cytomorphological criteria, in Giemsa-stained slides more CTC were identified as in IF stained slides in 22/29 patients (76%) (**Figure 2A**). In 5/29 patients (17%), more CTCs were detected via the IF staining protocol. In 2/29 patients (#9 and #27; 7%) no CTCs were detected using either of the staining protocols (**Figure 2A**). Overall, 3 fold more CTCs were detected via the Giemsa staining protocol (**Figure 2B**). Bland–Altman analysis revealed a mean difference of approximately 8.5 units (bias) between Giemsa stain and IF stain, with Giemsa stain yielding higher cell counts (**Figure 2C**). The 95% limits of agreement ranged from –20 to + 40 units (**Figure 2C**). Notably, the difference between methods was low at lower average and increased with increasing average of both methods (**Figure 2C**). The two methods are equivalent at low averages, but not across all measurement range, especially at high averages. Among all the CTCs detected via the IF staining protocol, which allowed discrimination between the phenotypes of the CTCs, 81% were in the M phenotype, 19% of CTCs in the hybrid E/M phenotype and 0% in the E phenotype (**Figure 2D**). However, the E/M CTCs detected were identified in a single patient (1/29, 13; 3%) (**Figure 2E**); in the remaining patients whose CTCs were identified via IF staining, the cells were in the M phenotype (19/29, 66%) (**Figure 2E**).

### 3.4 Morphological features of metastatic breast cancer CTCs in Giemsa- or IF-stained slides

**Figure 3:**
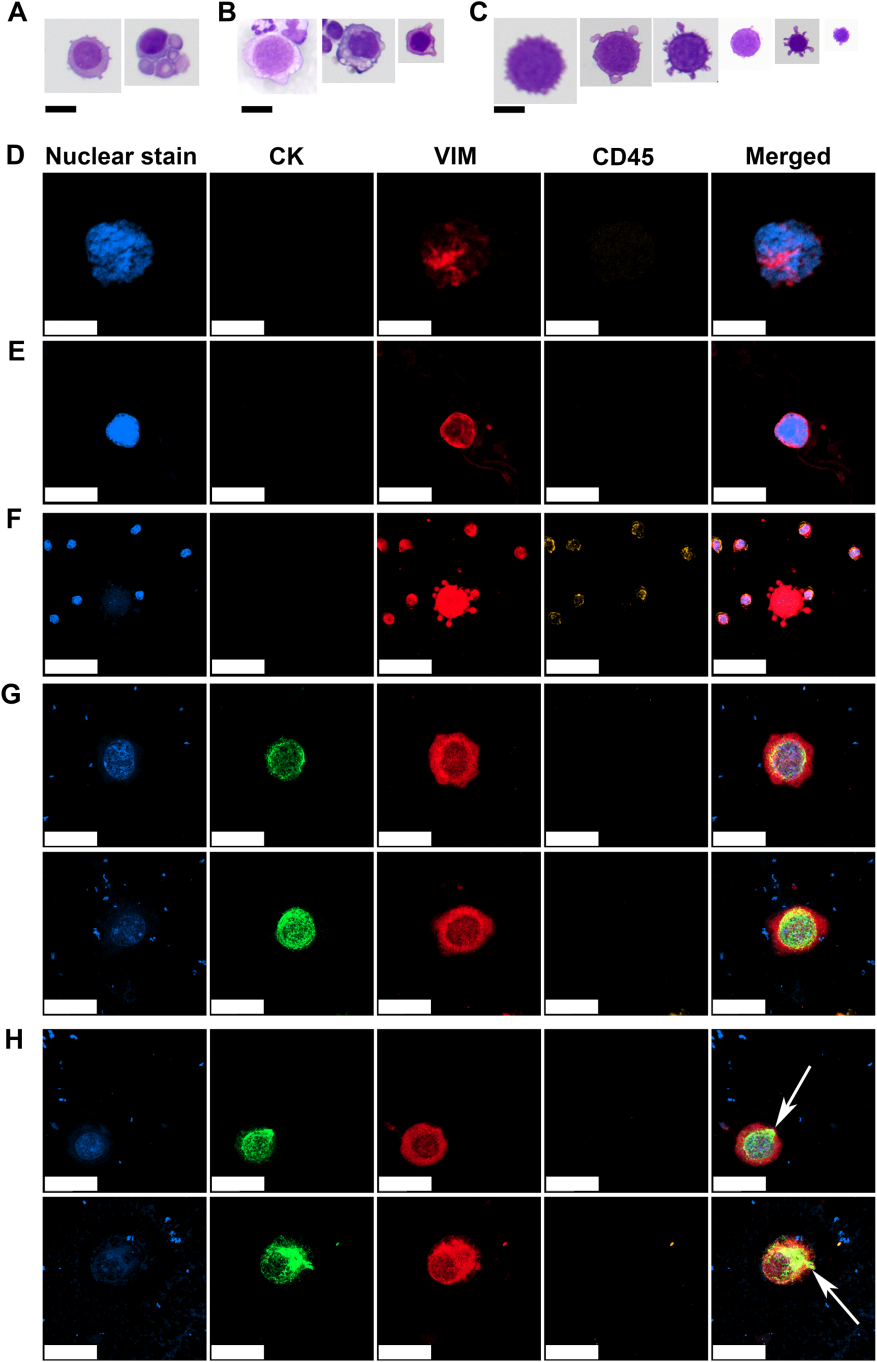
Representative morphologies of CTCs observed on Giemsa-stained and IF slides. Morphologically preserved malignant cells with large nuclei, scant basophilic cytoplasm, high N/C ratios, visible nucleoli, and intact chromatin structures **(A)**. Degenerated malignant cells showing cytoplasmic vacuolization, membrane blebbing, and an irregular nuclear membrane but with retained nuclear detail **(B)**. Severely degenerated CTCs with heterogeneous sizes, indistinct N/C borders, and condensed, homogenized intracellular contents **(C)**. Mesenchymal CTC with a large nucleus, coarse to clumped chromatin, indistinct N/C borders, displaying a VIM^+^/CD45^−^ immunophenotype **(D)**. Mesenchymal CTC with a large nucleus, fine chromatin, and poorly defined N/C boundaries, also showing a VIM^+^/CD45^−^ immunophenotype **(E)**. Degenerated mesenchymal CTC with faint or nearly absent nuclear signal, indistinct N/C borders, and prominent cytoplasmic blebbing, retaining a VIM^+^/CD45^−^ immunophenotype indicative of apoptotic or necrotic progression **(F)**. E/M CTCs with oval nucleus, coarse chromatin, perinuclear CK staining, cytoplasmic VIM staining and negative CD45 staining **(G)**. Subset of E/M CTCs displaying polarized CK signal extending from the perinuclear region to the cell periphery (white arrows) **(H)**. CK, cytokeratin; VIM, vimentin; CD45, cluster of differentiation 45/leukocyte common antigen. Scale bar = 20 µm in **(A-D, G, H)** and 50 µm in **(E, F)**.

The Giemsa and IF-stained slides containing CTC samples from the patient cohort were analyzed and CTCs were characterized based on their morphological features. The CTCs identified in this patient cohort exhibited marked cytomorphological heterogeneity. Based on Giemsa-stained cytospin preparations, three distinct cytological subpopulations were classified according to their nuclear and cytoplasmic features. The first population comprised well-preserved malignant cells with cytomorphological characteristics of epithelial tumors, resembling those observed in FNAB (17). These cells presented large, round to oval nuclei with a high N/C ratio, scant basophilic cytoplasm, well-defined nuclear contours, visible nucleoli, and preserved chromatin structure (**Figure 3A**). These morphologically intact cells were detected only in the samples from one patient (#13) displaying CTCs with an E/M phenotype (**Figures 2C, D**). The second population presented features consistent with degenerating malignant cells. These cells retained several characteristics of the first group such as large nuclei, a high N/C ratio, and a visible chromatin structure. However, they also displayed cytoplasmic vacuolization, irregular nuclear membranes, and disrupted plasma membranes with membrane blebbing and surface villi (**Figure 3B**). The third population was morphologically the most diverse, with cell diameters ranging from 16 to 35 µm (**Figure 3C**). These cells exhibited severe cytoplasmic and nuclear degeneration, with markedly irregular and disrupted plasma membranes, frequent blebbing, and villi (**Figure 3C**). The nuclear borders were indistinct or completely effaced, and the nucleocytoplasmic boundary was poorly detectable. The intracellular contents appeared homogenized and hyperchromatic, which was consistent with advanced degenerative or necrotic changes, including karyolysis and karyorrhexis (**Figure 3C**).

IF staining supported the identity of CTCs previously identified on Giemsa-stained slides by employing a marker-based approach. CTCs displaying the M phenotype exhibited variable nuclear and cytoplasmic features but consistently demonstrated positivity for VIM and negativity for the leukocyte common antigen CD45, supporting their nonhematopoietic origin. One subset of CTCs presented large nuclei with coarse to clumped chromatin and indistinct N/C borders (**Figure 3D**). Another subset displayed large nuclei with fine chromatin and poorly defined N/C borders (**Figure 3E**). A third subset consisted of more degenerated cells with faint or nearly undetectable nuclear signals, indistinct N/C borders and prominent cytoplasmic blebbing (**Figure 3F**), suggesting cell death-associated changes in M-type CTCs.

CTCs exhibiting a hybrid E/M phenotype were identified in the sample of one patient (#13) who also demonstrated well-preserved malignant CTCs on Giemsa-stained slides (**Figure 3A**). These E/M-phenotype CTCs displayed round to oval nuclei with clearly visible, coarse to clumped chromatin, a perinuclear CK signal, cytoplasmic VIM expression, and were negative for CD45, confirming their E/M and nonhematopoietic profiles (**Figure 3G**).

Notably, a subset of CTCs demonstrated a distinct pattern of CK polarization, extending from the perinuclear region toward the cytoplasmic periphery (**Figure 3H**, white arrows). This spatial redistribution of CK suggests a transitional cellular state, possibly associated with Epithelial-to-Mesenchymal Transition (EMT) during intravasation. The patient with hybrid CTCs shown in **Figure 3H** (#13) was a 52-year-old woman, which was diagnosed with Invasive Lobular Carcinoma (ILC) and Invasive Ductal Carcinoma (IDC) of NST, stage cT2N3M0, HR+, HER2-negative, four years prior to donating the CTC sample. She received neoadjuvant chemotherapy with anthracyclines and taxanes, followed by breast-conserving surgery and axillary lymph node dissection. Pathological evaluation revealed residual ILC (ypT1a ypN3, with 14 positive lymph nodes) and residual IDC (ypT1b ypN0) NST. Adjuvant treatment included radiotherapy, letrozole, and zoledronic acid (administered every six months). Approximately 2.5 years after diagnosis, the patient developed bone and bone marrow metastases. She was subsequently treated with two lines of chemotherapy (capecitabine and doxorubicin), followed by endocrine therapy with fulvestrant and abemaciclib. At the time of CTC analysis, the patient exhibited overt visceral disease progression, including bilateral pleural involvement with symptomatic effusions, peritoneal carcinomatosis with ascites, and right sided hydronephrosis. She was treated with weekly paclitaxel. Two months later, she developed diffuse cranial leptomeningeal disease. The patient passed away 3.5 months after CTC sampling.

To determine whether the observed CK polarization could be an artifact introduced during sample processing, MCF7 cells were subjected to the same enrichment and staining protocol. IF analysis revealed no evidence of polarized CK distribution in MCF7 cells, either before or after the isolation procedure (**Supplementary Figure 1**), indicating that the CK pattern observed in patient-derived CTCs is unlikely to be a result of the isolation method.

## 4 Discussion

In this study, we developed an IF staining protocol for the detection and characterization of CTCs in peripheral blood samples from patients with metastatic breast cancer. The protocol utilized antibodies against CK epithelial markers, a VIM mesenchymal marker, nuclear Hoechst 33342 staining and CD45 as a negative selection marker for hematopoietic cells. A direct comparison of CTC detection rates and morphological details was made between IF-stained slides and routine Giemsa-stained slides. Interestingly, Giemsa staining enabled the detection of a higher number of CTCs compared to our IF protocol. Lower detection rate in IF slides could result from the loss of fragile or loosely adherent cells due to the more complex staining procedure, especially the methanol fixation step. Additionally, in IF-stained samples, we observed that CTCs presented faint nuclear signals, potentially impairing their recognition. Nevertheless, IF staining supported the identity of CTCs identified via Giemsa staining by employing a marker-based approach and allowed detailed phenotypic discrimination and structural analysis of CTCs, including the identification of a distinctive CK distribution pattern suggestive of a transitional state during intravasation. Both CTC detection methods therefore have their respective advantages and limitations, which are summarized in **Table 2** based on various performance parameters.

**Table 2:** Comparative parameters of Giemsa- and IF-staining of CTCs highlighting advantages and limitations of both methods.

Parameter	Giemsa staining	IF staining
Detection rate	High (approximately 8.5-times more CTCs detected in each sample (Bland–Altman analysis)	Moderate (cell loss during methanol fixation step, possible underestimation of CTC number due to low nuclear signal in some CTCs)
Specificity	Moderate (lacks antigen specificity; relies solely on morphology)	Moderate (detects multiple target markers, may pose a challenge as some cells may express low or variable levels of CK and VIM)
Operator dependence	High (requires significant experience to interpret cell morphology accurately due to morphological complexity, introducing subjectivity and potential variability across observers)	Moderate (requires experience in fluorescence microscopy for distinguishing the autofluorescence from the fluorescent signal)
Equipment	Standard bright-field microscope sufficient	Requires fluorescence microscope and image acquisition software
Resolution and detail	Moderate (useful for assessing morphology and nuclear details)	High (spatial resolution for subcellular localization such as CK polarization)
Time to result	Shorter (standardized short staining procedure and immediate analysis)	Longer (involves antibody incubation, washing and delay of imaging due to antifade mounting)
Cost	Low (dye staining and bright-field microscope)	Moderate (antibodies and fluorescence imaging systems)

While ctDNA analysis is steadily progressing toward clinical standardization, the detection of CTCs remains technically challenging due to heterogeneous isolation and detection methodologies, limiting its broader diagnostic and clinical application (7, 8, 23). To date, the CellSearch^®^ system remains the only Food and Drug Administration (FDA)-approved and clinically validated platform for CTC enumeration in metastatic breast, prostate, and colorectal cancer, where the CTC count is recognized as a prognostic marker (24, 25). This system uses a fully automated immunomagnetic enrichment and IF-based detection method (24). In contrast, most other platforms rely mainly on individual detection technology and interpretation of the data obtained. The Parsortix^®^ system, which was employed in our study, is FDA-approved for the isolation of CTCs from patients with metastatic breast cancer (26, 27). However, the identification and interpretation of CTCs post-isolation remain largely manual and rely on researcher expertise. Our group developed a CTC detection method based on cytomorphological criteria following routine cytological Giemsa staining (15). To further confirm the identity of the detected CTCs, an IF-based detection method was developed in the current study. The IF-based detection method confirmed the identity of the CTCs identified by Giemsa staining via marker-based characterization and morphological features; however, the detection rate was lower, probably because of processing-related cell loss during the more complex staining procedure. Furthermore, subpopulations of CTCs M presented with faint or almost undetected nuclear signals, potentially arising from ballooning degeneration of cells or karyolysis and karyorrhexis, resulting in anuclear necrotic cells (28). These processes can reduce the detection sensitivity as the identification of CTCs relies mainly on the fact that tumor cells contain a large nucleus (29, 30). This could pose a major problem in automated image analysis systems that rely solely on the fluorescence images (31, 32).

Despite certain limitations, the IF-based detection approach provides enhanced specificity and enables detailed phenotypic and morphological characterization of CTCs. Notably, we observed a distinct polarized distribution of CK in IF-stained CTCs, which may indicate a transitional state of tumor cells during intravasation into the bloodstream. MCF7 cells processed via the same isolation and staining protocol as clinical samples demonstrated that the polarized CK pattern observed in patient-derived CTCs is not an artifact of the isolation procedure. The CK network has emerged as a critical regulator of EMT and metastatic progression (33). In the epithelial state, CKs form a filamentous network that spans the cytoplasm from the nucleus to the cell membrane, providing structural integrity and resistance to mechanical stress (34). In our study, CK staining was predominantly perinuclear, suggesting a reorganization of the CK network that aligns with a migratory phenotype. This pattern mirrors the cytoskeletal rearrangements observed in the sphingosylphosphorylcholine-induced transition of CK in PANC-1 pancreatic cells, where CK redistribution to the perinuclear region increased motility of cells (35). Such reorganization enhances the capacity of cells for directed migration and environmental responsiveness, underscoring the role of CKs in supporting invasive behavior (33, 36).

However, the polarized CK pattern we observed has not, to the best of our knowledge, been previously reported in CTCs, highlighting a potentially novel aspect of CK dynamics in the context of CK cellular organization in cellular polarization in the liquid phase. The polarity of cells during liquid or detached phases and the relevance of such polarization for metastasis have yet remained unclear. A distinct type of polarity termed Single-Cell (sc) polarity as a type of polarity that cells adopt in the liquid phase without directional stimulus was characterized by a round morphology of cells and the formation of a pole containing ezrin, F-actin, myosin light chain, phosphatidylinositol 4,5-bisphosphate, cell adhesion molecules, integrins and plasma membrane folding (37). The authors concluded that in circulation, the cells may acquire sc polarity via further mechanisms. CK reorganization appears to be a viable mechanism, as the keratin network in the cell is responsible for mechanical resistance.

The assembly of the keratin cytoskeleton in polarized cell protrusions was demonstrated in embryonic Xenopus mesendoderm cells in response to the tension to cadherin-based adhesions via magnetic tweezers. The mechanical force applied locally to C-cadherins on single Xenopus mesendoderm cells was sufficient to induce the reorganization of the keratin network toward these stressed sites inducing polarized cell protrusions and persistent migration typical of individual cells within a collectively migrating tissue (38).

A polarized distribution of CK was observed in one patient who was diagnosed with lobular breast carcinoma. At the time of CTC sampling, the patient exhibited fulminant disease progression involving multiple visceral serous membranes, including the pleura and peritoneum. Notably, two months later, disease dissemination extended to the meningeal membranes in the central nervous system. These observations suggest that the presence of such CTCs may be indicative of a distinct pattern of metastatic progression via serous membranes. However, this phenomenon has been observed in only one patient thus far and needs to be further explored in the future. With recent technological advancements enabling more reliable isolation and characterization of CTCs, future clinical studies should also address CK polarization together with clinical patient characteristics in greater detail.

This study has several limitations that should be considered when interpreting the findings. First, the small cohort of patients restricts the generalizability of the results and the ability to draw clinical correlations, including associations between CTC characteristics and patient outcomes. A larger sample size would be necessary in order to validate the findings. Second, the limited cohort size also influenced the range of CTC phenotypes detected. In our analysis, we did not observe any pure E phenotype CTCs; only one patient exhibited E/M hybrid phenotype, and the remaining CTCs were classified as M. As a result, the morphological and phenotypic assessments in this study did not encompass the full spectrum of CTC states known to exist in breast cancer, which may reduce the applicability of our conclusions to broader patient populations. Third, our study lacks the molecular validation, such as single-cell RNA sequencing, to confirm the malignant origin of the cells identified as CTCs. Due to the limited number of enriched cells and the absence of optimized protocols for RNA extraction from single cells in our laboratory, we were unable to perform such analyses. Future studies should aim to incorporate sc transcriptomic approaches to provide deeper insights into the heterogeneity and tumor-specific signatures of CTCs.

The last limitation is that the complexity of the IF staining protocol likely contributed to cellular loss during sample processing. In the first methanol fixation step, the slides are placed in a glass container filled with ice-cold methanol for several days until the staining procedure (to collect a cohort of slides). We were interested in the contribution of cell loss during this step. For this purpose, we conducted an experiment with a defined number of cells on the slides (cytospin of three concentrations of MCF7 cell suspension) and compared the degree of cell loss after air-dried fixation and methanol fixation, followed by the same Giemsa staining procedure. We found significant cell loss when methanol fixation was used compared with air-dried fixation, with approximately 30% more cell loss on average (**Supplementary Figure 2**). The methanol fixation step can therefore be considered one of the most important steps in further protocol optimization. One of the possible alternatives would be ice-cold acetone fixation and the storage of dry slides at freezing temperatures until staining. The multiple incubation and wash steps inherent to the IF procedure may have additional effects on the loss of fragile or loosely adherent CTCs, thereby underestimating the total CTC burden. To address this, several optimization strategies should be considered to improve cell retention while maintaining staining specificity and signal intensity. Furthermore, the study employed a limited antibody panel, which may not fully capture the phenotypic heterogeneity of CTCs. Future optimization of the IF-staining protocol should include a broader range of markers to enable more comprehensive characterization of CTC subpopulations. One potential strategy involves the use of directly labeled primary antibodies, which could eliminate the need for secondary antibody incubation and reduce the number of wash steps (39). However, this approach typically results in weaker signal intensity, which may be insufficient for the reliable detection of rare and heterogeneous CTCs. More advanced techniques, such as polymer-based fluorophore conjugates, brighter and more photostable dyes, or enzymatic amplification methods, such as tyramide signal amplification, may offer improved signal enhancement while minimizing cell loss (40, 41). Additionally, protocol modifications that reduce the number of wash steps could be explored, although this carries the trade-off of increased nonspecific background fluorescence, potentially compromising image clarity and method specificity.

Comparing our findings with those of other studies is challenging due to substantial methodological variability, including differences in blood sample collection protocols, CTC enrichment or capture techniques, and detection methods such as immunocytochemistry, IF, or molecular assays. Despite these differences, a comparison can be drawn based on the detection rates of CTCs in patients with metastatic breast. A recent study in patients with metastatic breast cancer comparing two widely used CTC enrichment platforms, CellSearch and RareCyte, reported CTC detection in 65% and 75% of paired patient samples, respectively, using 7.5 mL of blood (19). In contrast, our study detected CTCs in 27 out of 29 patients (93%) using a 10 mL blood volume. The comparator study reported CTC count ranges of 0 to 2289 for CellSearch and 0 to 1676 for RareCyte, while our CTC counts ranged from 0 to 48 using detection via Giemsa staining and up to 23 via IF staining. Although our workflow identified fewer CTCs overall, the number of patient samples analyzed was substantially lower (29 *vs*. 100), which limits the direct comparability of CTC count distributions between studies. The study populations also differed substantially. The comparator trial enrolled female patients with metastatic breast cancer who were about to begin their first-line systemic treatment. In contrast, our cohort consisted exclusively of patients receiving second-line or later chemotherapy with radiologic and/or laboratory-confirmed disease progression; and we excluded patients on first-line therapy. These differences in treatment lines likely contributed to the variation in CTC numbers reported by the two studies. When comparing our results to a study that employed the same CTC capture platform (Parsortix) combined with either IF or cytopathological staining, more comparable outcomes were observed (18). In that study, 76 patients with metastatic breast cancer were enrolled, and up to 10 mL of peripheral blood was processed per patient. While the treatment line was not specified, CTC detection rates were 42.9% using cytopathological staining (with a range of 0–41 cells per sample) and 45% using IF (range: 0–125 cells per sample). These findings show methodological and technical similarities with our approach, while our overall detection rates were notably higher, the range of detected cells per sample was more in accordance to our study.

In conclusion, Giemsa and IF staining each offer distinct advantages for CTC detection in patients with metastatic breast cancer, with Giemsa enabling broader morphological assessment and IF providing marker-specific precision. From a clinical perspective, an integrative strategy that combines both Giemsa and IF techniques may offer a more comprehensive view of CTC populations in patients with metastatic breast cancer, thereby enhancing diagnostic precision. Moreover, we observed a polarized distribution of CK in a patient who experienced fulminant disease progression with involvement of multiple visceral serous membranes. This observation underscores the heterogeneity of CTC phenotypes and warrants further investigation to elucidate its potential clinical and prognostic significance. Further investigations and interdisciplinary collaboration among clinical oncologists, clinical pathologists, cancer immunobiologists, translational immunobiologists, health-system coordinators, and others are warranted to advance this field.

## Data Availability

The original contributions presented in the study are included in the article/**Supplementary Material**. Further inquiries can be directed to the corresponding authors.
