# Supplementary material for: Complementary detection strategies for circulating tumor cells in breast cancer: clinical implications of combining immunofluorescence and cytopathological staining
Source: Front Oncol. 2025 Jul 23;15:1632245. doi: 10.3389/fonc.2025.1632245 (PMC12325023; doi:10.3389/fonc.2025.1632245)
Supplement: Supplementary file 1 [file DataSheet1.pdf]

Supplementary Material

1.1 Supplementary Figures

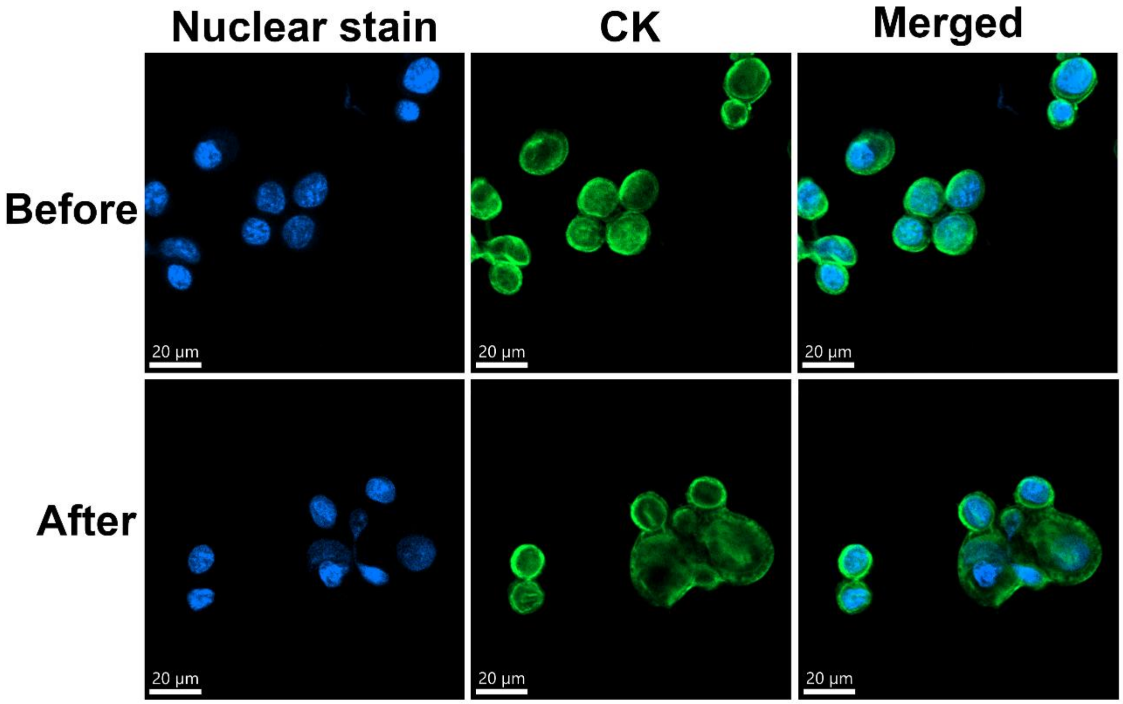

Supplementary Figure 1. Control Experiment of evaluation of CK distribution in MCF7 cells following isolation protocol. MCF7 cells processed using the same isolation and staining protocol as clinical samples. No CK polarization pattern was observed before or after the isolation process. All MCF7 cells showed uniform CK distribution, confirming that the polarized CK pattern seen in patient-derived CTCs is not an artifact of the isolation procedure. CK: cytokeratin, Before: MCF7 cells before isolation, After: MCF7 cells after isolation. Scale bar = 20 µm.

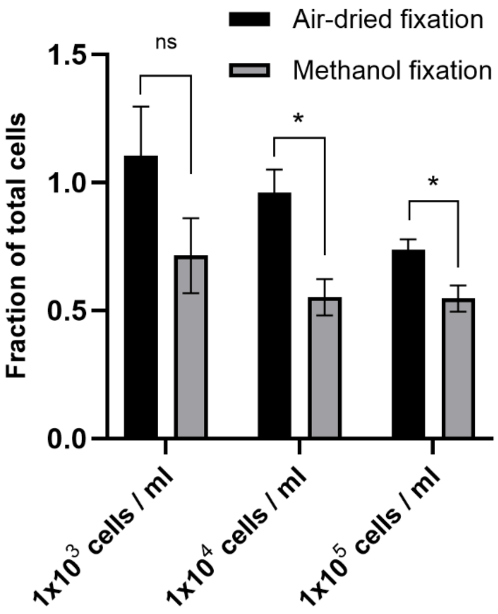

Supplementary Figure 2. Loss of cells after air-dried or methanol fixation. Three concentrations of MCF7 cells were prepared (1×10^3 cells / ml, 1×10^4 cells / ml and 1×10^5 cells / ml) and 6 cytospins were prepared from each cell suspension. Three of them were air-dried and stained with a standard Giemsa stain. The other three were fixed in methanol and 4 days after were stained with the same Giemsa protocol. The slides were scanned using NanoZoomer slide scanner (Hamamatsu, Hamamatsu, Japan) and the number of cells was quantified by QuPath open source software using Cell detection function. The number of cells after each fixation method was normalized to total cell number added into the cytofunnel.
